# Systematic Review of the Application of Artificial Intelligence in Healthcare and Nursing Care

**DOI:** 10.21315/mjms2024.31.5.9

**Published:** 2024-10-08

**Authors:** Thai Hau Koo, Andee Dzulkarnaen Zakaria, Jet Kwan Ng, Xue Bin Leong

**Affiliations:** 1 Department of Internal Medicine, Hospital Universiti Sains Malaysia, Kelantan, Malaysia; 2 Department of Surgery, Hospital Universiti Sains Malaysia, Kelantan, Malaysia; 3 School of Medical Sciences, Universiti Sains Malaysia, Kelantan, Malaysia

**Keywords:** systematic review, artificial intelligence, healthcare, nursing care, ethical considerations, regulatory frameworks

## Abstract

This systematic review explores the complex relationship between artificial intelligence (AI) and healthcare, with an explicit focus on nursing care. Examining a range of studies from 2020, the research investigates the impact of AI on clinical decision-making, patient care and healthcare administration. Through a comprehensive literature review, the study highlights the potential benefits of AI integration in improving the efficiency and efficacy of healthcare. AI technologies offer opportunities for personalised patient care, predictive analytics and enhanced clinical processes, with the ultimate aim of transforming the healthcare system. However, ethical considerations and regulatory frameworks are crucial, emphasising patient privacy, autonomy and data security. The findings underscore the need for transparency, accountability and fairness in the application of AI in healthcare. While AI promises to improve patient outcomes and streamline healthcare delivery, careful consideration of ethical implications and regulatory compliance are essential for responsible implementation.

## Introduction

Artificial intelligence (AI) has transformed the healthcare and nursing industries by improving patient care, clinical decision-making and the quality of healthcare. The integration of AI into nursing and healthcare systems has the potential to improve quality and achieve better patient outcomes and healthcare efficiency. However, healthcare costs continue to grow due to an aging population and chronic diseases. Finding new solutions to improve the accessibility, cost and quality of healthcare is crucial, given the various difficulties faced by healthcare systems worldwide. AI is transforming healthcare by automating processes, analysing data and providing insights to practitioners. It is revolutionising imaging and diagnosis through advanced analysis of computed tomography (CT), magnetic resonance imaging (MRI) and X-ray scans. These technologies have been shown to easily detect outliers (1). Machine learning algorithms enhance practitioners’ capabilities, particularly in medical image analysis, enabling early disease detection and alternative therapies (1).

AI and predictive analytics have the potential to revolutionise how healthcare decision-makers utilise data. Healthcare practitioners are using cutting-edge technologies to evaluate patients and make precise estimates of the healthcare management. These algorithms can detect patterns and trends in health data, including genetics and electronic health records, that clinicians may overlook. In healthcare, AI algorithms can improve decision-making, treatment regimens, clinical care and patient outcomes; increase patient engagement and track individual health and well-being (2). Chatbots and virtual health assistants employ natural language processing (NLP) to help people book appointments, obtain specialised health information, and provide remote monitoring and care. AI can enhance health and well-being management by providing practical solutions that transform both nursing practices and overall healthcare delivery. Prioritising patient confidentiality, independence, and well-being during the development and deployment of AI technologies is crucial (3).

Healthcare technology has advanced through the integration of AI into nursing care and healthcare systems. The exponential growth of healthcare data and the complexity of patient care offer new opportunities for employing AI to enhance healthcare delivery systems, clinical decision-making and patient outcomes. This comprehensive study investigates the application of AI in healthcare and patient search systems, focusing on AI’s advantages, challenges, and implications for healthcare and nurses (4).

AI research involves the development of algorithms and computational methods that mimic human intelligence. The goal is to boost the routine intelligence and efficiency of computers. The integration of AI has the potential to disrupt healthcare. This area includes patient interaction, predictive analytics, medical imaging, diagnostics and personalised medicine. Modern deep learning and machine learning algorithms allow for lightning-fast and pinpoint accuracy data analysis of healthcare datasets (5). Early identification of disease and optimisation of treatment are possible through proactive health monitoring. Despite its immense potential, the application and use of AI in the healthcare industry faces challenges and ethical dilemmas (6). The use of AI in healthcare must be considered ethically in several ways. In this study, we discuss the implications for healthcare workers, including concerns about algorithmic bias, data privacy and security, as well as regulatory compliance. Additionally, we review the use of AI in healthcare and nursing.

## Method

### Research Design

**Figure 1:**
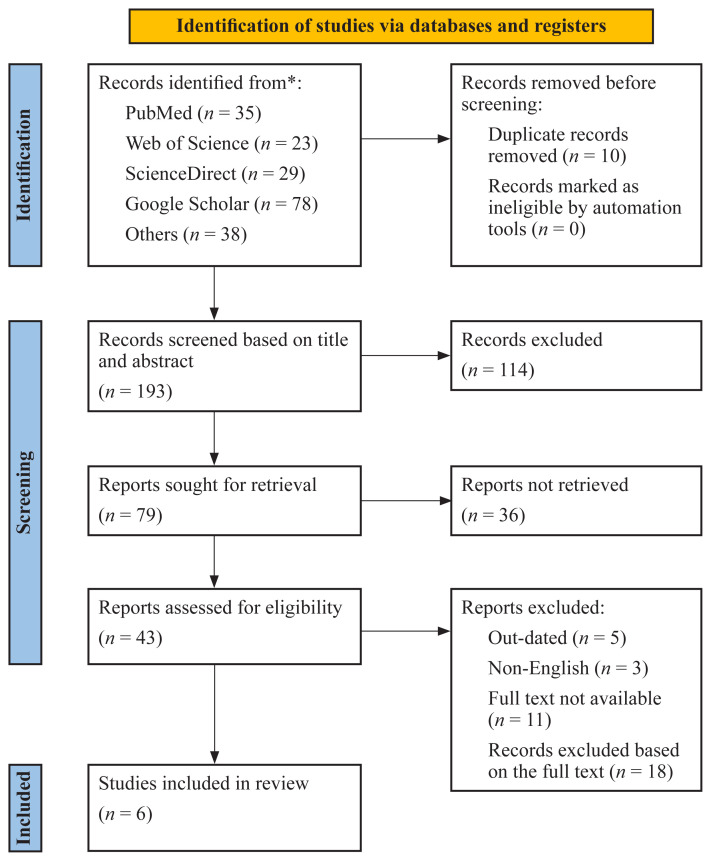
This is a figure of the PRISMA chart for quantitative analysis (9)

The study follows the Preferred Reporting Items for Systematic Review and Meta-Analysis (PRISMA) methodology (Figure 1) to conduct qualitative and quantitative analyses to investigate the impact of AI on clinical decision-making, patient care and healthcare administration (7).

### Selection Criteria

The selection of relevant articles was carried out by initial screening based on inclusion and exclusion. The inclusion criteria consisted of peer-reviewed articles published in English between January 2020 and December 2023 that presented qualitative and quantitative analyses and discussed the latest advances in AI use in the nursing and healthcare sectors. Exclusion criteria were studies that fell outside the specified time interval, and studies on AI use that were unrelated to nursing and healthcare outcomes.

### Search Strategy

A search for peer-reviewed papers was conducted in the following electronic databases: PubMed, IEEE Xplore, Elsevier, Web of Science, ScienceDirect and Google Scholar. The search encompassed the period from January 2020 to December 2023. We also screened the references and citations of the published papers for possible additional studies. Only studies published in English were included in the review. Search strategies were developed using keywords, their synonyms, abbreviations and MeSH terms for ‘artificial intelligence,’ ‘machine learning,’ ‘healthcare’ and ‘nursing care’ with Boolean operators (AND, OR) to construct comprehensive search strings. The inclusion of asterisks (*) improved the literature retrieval. The search strategy was checked and maximised in PubMed and replicated in other databases using appropriate search agreements.

### Study Selection

After a preliminary search, the search results were thoroughly screened using systematic review criteria to identify relevant studies. The studies were then selected through literature searches, followed by three rounds of screening and filtering. In the first stage of the pilot review, all retrieved papers were entered into Mendeley software (1.19.4–win32) and an initial de-duplication of studies was performed. Two independent reviewers (JKN and XBL) independently screened the titles and abstracts and retrieved the full text and supplementary files according to the above eligibility criteria using Rayyan software. The corresponding authors of the relevant articles were contacted by email to obtain missing information. Only articles for which full text was available were included in this review.

### Data Collection Process

All included articles were reviewed, screened, extracted and summarised according to their primary classifications by two independent reviewers (JKN and XBL) and then saved as Microsoft Word and Excel files to simplify the filtering process. The authors read the full text of every paper. We were able to develop the proposed taxonomy using a variety of highlights and comments on the papers studied as well as a running classification of all the articles. Remarks were recorded either on paper or electronically, depending on the writing style of each author. This was followed by a further procedure to characterise, describe, tabulate, and draw conclusions about the key findings. Data from the studies were collected and analysed to provide a complete review of the literature on AI in healthcare and nursing.

## Results

The systematic search strategy generated a complete database of 203 articles from PubMed (*n* = 35), Web of Science (*n* = 23), ScienceDirect (*n* = 29), Google Scholar (*n* = 78) and others (*n* = 38); the search process and the results of the selection are shown in Figure 1. In addition, we used a rigorous screening process that eliminated 10 duplicates as well as 114 articles that we considered irrelevant to our study focus. Thirty-seven studies were excluded because they did not meet the predefined inclusion criteria. Six eligible studies were reviewed according to eligibility criteria.

AI has improved healthcare research and practice. It strives to enhance healthcare administration, patient care, and clinical judgment. According to Ben-Israel et al. (8), machine learning enhances patient healthcare by streamlining administrative procedures and reducing operational complexities within the healthcare system (Table 1). Healthcare workers can utilise machine learning algorithms to improve patient outcomes by enhancing the patient experience, accurate diagnosis and customised therapy. The growing healthcare industry can benefit from the findings of Chen et al. (3) on AI in nursing and healthcare administration. According to their article, AI must overcome challenges to meet the growing demands of healthcare systems globally. Several challenges need to be overcome before AI can fully realise its full potential in healthcare delivery. Healthcare workers can employ AI technologies to improve patient care, simplify procedures, and streamline operations (3). Chen and colleagues (3) hypothesised that integrating AI into healthcare will necessitate revising nursing care management goals to address potential conflicts. They concluded that tailored healthcare technology is essential and meaningfully discussed the pros and cons of healthcare AI.

Data-driven AI can potentially improve IT decision-making in health and social care, according to Cresswell et al. (4). Their extensive study highlights the potential of AI to improve healthcare decision-making. However, potential challenges posed by algorithmic biases and poor data need to be explored to address the complex issue of quality of care. When adopting AI-powered decision-support technologies, healthcare institutions should be mindful of fairness and honesty. Industry leaders, data scientists and policymakers need to work together to optimise AI in healthcare decision-making. Kaieski et al. (9) conducted an extensive literature review to bridge knowledge gaps in the application of AI techniques for vital sign studies in hospitalised patients. The extensive literature review demonstrates the great potential of AI for early clinical diagnosis and intervention. AI analytics can help healthcare professionals by analysing physiological data. Drug efficacy and patient outcomes have dramatically improved using this technique. To realise its full potential in clinical decision support and vital signs monitoring, AI must overcome many challenges, including understanding, characterising models and integrating diverse data sources (4).

Research by Ng et al. (6) showed that AI-assisted medication can improve nursing practice and patient outcomes. AI-integrated clinical nursing care improved according to specialists. Ng et al. (6) identified multiple AI-based nursing care applications that could improve nursing care in various healthcare settings. AI can enhance healthcare by automating tedious tasks and creating individualized treatment plans for each patient. However, it is necessary to thoroughly investigate the ethical ramifications and staff training required to effectively integrate AI into nursing practice. The study by Seibert et al. (5) provided valuable insights into the application of AI in nursing care, examining the potential advantages and disadvantages of AI-powered nursing solutions. The researchers highlighted industry trends and game-changing opportunities, focusing on predictive analytics and remote patient monitoring prospects. They emphasised that AI is crucial to the optimisation and improvement of healthcare. After evaluating massive amounts of real-world data, the researchers gained valuable insights into AI and nursing care (5).

Sharma et al. (1) discussed the current state of AI and analysed its applications in healthcare, including diagnostic and therapeutic support. Their findings highlight the multiple applications of AI in healthcare settings and identify potential areas for further investigation, such as enhancing diagnostic accuracy, optimising treatment plans and improving patient monitoring. Despite concerns related to algorithmic bias and data privacy, the study suggests that AI can improve clinical operations and patient outcomes. Von Gerich et al. (2) conducted a comprehensive review of the literature on AI-driven nursing technologies, providing significant insights into the potential benefits and drawbacks of using AI in nursing care. Significantly, AI technologies have changed nursing practice and improved patient care, with potential applications in robotics and decision systems. It is important to consider essential components such as data security, staff training, and nursing care monitoring for the appropriate integration of AI (1).

To assess the risk of bias across the studies, we used the Cochrane risk-of-bias tool for randomised controlled trials (RCTs) and then the Newcastle-Ottawa Scale (NOS) for observational studies. The study outcomes showed varying degrees of heterogeneity, with most of the studies having a low to moderate risk of bias. Significant biases were detected in the following areas: interruption of appropriate treatment, allocation of randomisation and masking of the treatment regimen.

## Discussion

### Implications for Healthcare Practice and Policy

The significant impact of AI on healthcare practice and policy requires detailed investigation. AI technologies can improve patient outcomes, healthcare delivery and operational efficiency (Table 2). AI has dramatically improved clinical decision-making in healthcare. AI-powered decision support systems can quickly provide advice and assistance to healthcare professionals. These systems need patient data to improve. Predictive analytics algorithms can identify potential patient challenges. Efficient diagnosis significantly improves patient outcomes and reduces risk, enabling healthcare providers to treat patients quickly. AI algorithms have the potential to improve healthcare by helping professionals diagnose and treat complicated illnesses. When personalised medicine is applied, the healthcare system can experience a paradigm shift, as treatments are customised to each patient. AI can evaluate clinical trial data, lifestyle decision data and genetic data (2).

Consider the potential influence of AI on clinical practice, healthcare administration and operations. AI-driven healthcare technologies have the potential to improve workflow and resource allocation, and NLP algorithms offer the potential to improve medical record documentation and coding. The medical team should prioritise patient care over administrative tasks. Hospitals can minimise waiting times and improve resource utilisation by predicting admission rates and regulating patient flow using predictive analytics algorithms.

Many people are concerned about the data privacy, legal repercussions and ethical issues that AI introduces to the healthcare industry. Policymakers must establish clear regulations and guidelines to ensure the ethical use of AI in healthcare while prioritising patient privacy and autonomy. Additionally, AI-powered decision-making requires transparency and accountability. AI technologies can also create healthcare disparities, so policymakers must act quickly to address algorithmic bias. For AI technologies to be effectively implemented in healthcare, it is crucial to emphasise a culture of collaboration and innovation (6).

### Challenges and Opportunities for AI Integration in Healthcare

If healthcare is to improve patient care, it must weigh the pros and cons of AI. The healthcare industry faces several challenges, including labour concerns, ethical dilemmas, new technologies and legislation. Two major barriers to AI integration in healthcare are compatibility of AI systems with existing healthcare infrastructure and insufficient data for AI model testing and training. Furthermore, the scattered nature of healthcare data can make it challenging to understand and use AI insights. Biases in data, lack of knowledge, and inconsistencies in data collection and management can lead to subpar outcomes by reducing the reliability and effectiveness of AI algorithms. To ensure the quality and reliability of advanced analytics data in healthcare, there is a need for facilities equipped with appropriate technology, rigorous control measures and standardised procedures (8).

AI algorithms applied to healthcare data can promote biases, which can worsen healthcare inequalities and treatment methods. The use of AI-powered decisions based on private medical data raises concerns about patient permission, privacy and autonomy. To maintain openness, accountability and fairness in the healthcare profession, it is essential to establish ethical standards and guidelines. These standards should involve health policymakers, healthcare practitioners and technology specialists (3).

### Ethical Considerations and Regulatory Frameworks for AI in Healthcare

The integration of AI in healthcare and nursing care raises ethical concerns that require robust regulatory frameworks (Table 2). Patient confidentiality, autonomy and privacy are essential when handling sensitive health data. Compliance with regulations such as the General Data Protection Regulation (GDPR) and the Health Insurance Portability and Accountability Act (HIPAA) is crucial to protect patient data and build confidence in AI-driven healthcare solutions (10). Additionally, uncertainty about accountability and AI system errors makes it challenging for healthcare professionals to evaluate AI performance (10). Algorithmic transparency and interpretability are necessary to maintain ethical standards and ensure patient safety when using AI in healthcare.

## Conclusion

The study examined AI in healthcare and nursing care, focusing on how AI can impact healthcare procedures, clinical judgement and patient care delivery. The ethical application of AI in healthcare raises legal and ethical concerns, where patient privacy, autonomy and fairness should be prioritised. Promoting honesty, accountability and safety is essential. Our extensive investigation shows that AI is improving the efficiency and effectiveness of diagnosis, treatment, personalised care, predictive analytics and clinical decision-making, benefiting both patients and frontline nurses. However, concerns regarding data privacy, transparency and algorithmic biases persist. To address these challenges, robust privacy protection, transparency and bias mitigation measures must be implemented, focusing on promoting ethical AI applications to improve patient safety and outcomes in healthcare.

## Figures and Tables

**Table 1:** Exploring the impact of AI on healthcare: Key findings from recent studies

Study (year)	AI applications	Key findings
Ben-Israel et al. (2020)	Machine learning	Improved patient healthcare delivery
Chen et al. (2022)	Healthcare management	Targeted discrepancies in the usage of AI techniques and their focus on health and nursing management
Cresswell et al. (2020)	Data-driven AI	AI benefits include decision-making and care coordination
Kaieski et al. (2020)	Vital sign analysis	AI’s ability to detect early progressions of some diseases or physiological changes could be an essential tool for clinical screening and interventions
Ng et al. (2022)	Clinical nursing care	AI’s importance in the advancement of the role of nurses in the clinical setting
Seibert et al. (2021)	Nursing care applications	Discoveries of AI in nursing care and future potential for this field
Sharma et al. (2022)	AI in healthcare	It is observed that AI has become an integral part of patient care in healthcare institutions
von Gerich et al. (2022)	Nursing technology	The medical care part based on AI and technologies used by nursing professionals alters healthcare delivery and medical decision-making

**Table 2:** Summary of the learning outcomes of this article

What was already known on the topic	What this study added to our knowledge
AI integration in healthcare is improving patient care	AI integration in healthcare, particularly nursing, can revolutionise patient care delivery and clinical decision-making
AI technologies offer opportunities for personalised care	This study highlights the ethical considerations and regulatory frameworks necessary for responsible AI implementation in healthcare
Predictive analytics and machine learning can enhance care	The systematic review underscores the importance of transparency, accountability and fairness in AI applications within healthcare
Ethical considerations and regulatory compliance are crucial for AI in healthcare	The study provides insights into the challenges and opportunities of AI integration in healthcare, emphasising the need to consider biases and data privacy concerns carefully
